# High efficient aging of sweet red wine by ultrasonic treatment and acoustic radical chemistry explanation

**DOI:** 10.1016/j.ultsonch.2025.107589

**Published:** 2025-09-27

**Authors:** Shiyao Wang, Xinyi Jia, Shuangli Xiong, Yisheng Chen

**Affiliations:** a College of Food Science and Engineering, Shanxi Agricultural University, Taiyuan 030801, China; b Cuisine Science Key Laboratory of Sichuan Province, Sichuan Tourism University, Chengdu 610100, China

**Keywords:** Ultrasonic aging, Sweet red wine, Sugar-acid ratio, Total phenols, Ultrasonic mechanism, Hydroxyl radicals

## Abstract

This study investigated the effects of low-power, low-temperature, and short-duration ultrasonic treatment on the quality of sweet red wine (SRW) to optimize flavor aging. Experiments were conducted by varying temperature (10–30 °C), duration (30–180 min), and power (100–300 W), with subsequent analysis of total sugar, acidity, tannins, esters, phenolic content, and antioxidant activity. Results demonstrated that low-power and low-temperature ultrasonic treatment effectively modulated the sugar-acid ratio in SRW. Tannin content exhibited minimal temperature dependence but was significantly influenced by ultrasonic power and duration, with duration exerting the strongest effect. Total ester content decreased with prolonged ultrasound exposure, though this reduction was mitigated under low-power conditions. Phenolic content declined as ultrasonic intensity increased. Sensory evaluation identified optimal aging parameters at 20 °C, 90 min, and 200 W. These results confirm that low-power, short-term ultrasound effectively ages sweet red wine while improving quality. This method offers a practical alternative to traditional aging, with potential benefits for wine production efficiency and quality control.

## 1 Introduction

Sweet red wine (SRW), a globally popular alcoholic beverage, is highly valued for its distinctive flavor profile and health-beneficial effects [1]. The production process typically involves two critical stages: grape fermentation and aging, with the latter being particularly crucial in determining the final quality characteristics of the wine [2]. Aging represents a complex biochemical process encompassing various physical, chemical, and biological reactions, including oxidation [3], esterification, polymerization, and volatilization [4]. These processes collectively contribute to significant modifications in the wine's flavor, aroma, texture, and color [5], ultimately yielding a product characterized by rich aromatic complexity, mellow taste, and well-balanced body. Notably, the transformation of monomeric tannins during aging reduces bitterness while enhancing the wine's fullness and structure [6].

Traditional aging methods, while effective, present substantial limitations in terms of time efficiency, labor requirements, and resource consumption, often conflicting with contemporary market demands [7]. Consequently, various artificial intervention strategies have been developed to accelerate the aging process, including oak product utilization, micro-oxygenation [8], ultrasonic treatment [9], and high-pressure processing [10]. Each method exhibits distinct effects on wine composition, with oak aging potentially leading to flavor homogenization [7] and micro-oxygenation risking excessive oxidation [8]. In contrast, physical aging techniques, particularly ultrasonic treatment, offer significant advantages in terms of process stability, safety, and environmental compatibility, aligning with modern food processing requirements through their chemical additive-free approach [11].

Ultrasound technology has emerged as an innovative non-thermal physical processing method [12], gaining widespread recognition in fermentation processes due to its equipment efficiency [13], low energy consumption, high processing efficiency, and minimal thermal impact. Ultrasonic treatment significantly enhances the flavor complexity of wine by promoting ester formation, increasing the content of phenolic and aromatic compounds, and improving phenol–protein interactions. Zheng et al. [14] reported a 24.59 % increase in total ester content following ultrasonic treatment (45 kHz, 360 W, 15 ℃, 30 min). While Celotti et al. [15] observed enhanced anthocyanin and phenolic compound concentrations with increased amplitude and treatment duration. Zhang et al. [16] elucidated ultrasound's role in modulating phenolic-protein interactions to improve sensory properties, and Liu et al. [17]Ultrasonic treatment (200 W, 20 °C, 80 min) increased the total phenolic content by approximately 12 % in the sweet fortified wine, while significantly enhancing key aroma compounds such as ethyl acetate and β-damascenone, thereby improving its overall flavor complexity. Sun et al. [18] reported that ultra-high pressure treatment (500 MPa, 20 °C, 30 min) promoted polyphenol polymerization and aroma integration in wine while preserving its non-color phenolic profile. The total ester content increased by approximately 15 %, accompanied by a reduction in off-flavor compounds such as aldehydes and ketones.

However, current ultrasonic applications in wine processing predominantly employ high-power and high-temperature conditions, which may compromise volatile compound integrity, accelerate undesirable oxidation processes, and potentially alter tannin structures, ultimately affecting flavor profiles and aging potential [12]. These limitations underscore the necessity for investigating low-power ultrasonic treatments under controlled temperature conditions, aiming to preserve SRW's sensory characteristics while enhancing its aging qualities [15].

This study systematically examines the effects of low-temperature, short-duration, and low-power ultrasound on SRW's physicochemical properties, with particular emphasis on flavor balance (total sugar and acid), ion concentration (conductivity), aromatic profile (total esters), and antioxidant capacity (total phenols, tannins, DPPH, and ABTS assays). By elucidating the mechanistic basis of ultrasound-induced aging at the free radical level, this research provides fundamental insights for optimizing ultrasonic parameters in SRW maturation processes.

## 2 Materials and methods

### 2.1 Wine samples and reagents

The 2024 vintage of North Ice Sweet Red Wine was purchased from Jilin Xue Co., Ltd., Tonghua City, Jilin Province, China, with an alcohol concentration of 10.0 % (v/v). The wine was fermented from fresh grapes. The wine samples were stored in separate polyethylene terephthalate (PET) containers (2.5 L each). Reagents used in this study, including copper sulfate, methylene blue, potassium sodium tartrate, formaldehyde, sodium hydroxide, glucose, concentrated hydrochloric acid, sulfuric acid, ethanol, tartaric acid, sodium benzoate, gallic acid, 1,1-diphenyl-2-picrylhydrazyl (DPPH), 2,2′-azino-bis(3-ethylbenzothiazoline-6-sulfonic acid) (ABTS), potassium iodide, and starch, were all analytical grade and purchased from Shanghai Aladdin Biochemical Technology Co., Ltd.

### 2.2 Ultrasonic optimization parameters

The ultrasonic aging technique was used to catalyze the samples. The experiments were conducted using an ultrasonic device (KS-500XDS, Tianjin Testing Instrument Co., Ltd., China) with a rated frequency of 40 kHz. A 250 mL volume of red wine was placed in a 500 mL stoppered wide-mouth bottle, and water was added to the ultrasonic bath to a level of 100 mm. Single-factor experiments were designed with power (100 W, 150 W, 200 W, 250 W, 300 W), temperature (10 ℃, 20 ℃, 30 ℃), and time (30 min, 60 min, 90 min, 120 min, 150 min, 180 min) as variable factors. These experiments were conducted to determine the preliminary processing parameters based on the assessment of drinking quality. Subsequently, response surface methodology was used to determine the optimal conditions to evaluate the effects of different ultrasonic power levels on the physicochemical indicators of SRW.

### 2.3 Experimental methods

#### 2.3.1 Determination of total sugar, total acid, and sugar-acid ratio

The determination method of total sugars was improved with reference to Yu [19] experimental method. Accurately pipetted 1 mL of the sample at 20 ℃ into a 50 mL volumetric flask. Since the total sugar content (TSC) in SRW is greater than 50 g/L, 5 mL of hydrochloric acid solution was added, followed by water to make up to 20 mL. The mixture was hydrolyzed in a water bath at 68 ℃ for 15 min and then cooled. The solution was neutralized with sodium hydroxide solution to neutrality and diluted to the mark with water. The volume of the sample consumed (V3) was recorded after replacing the glucose standard solution with the sample. The total sugar content (X1) was calculated using Eq. (1):

(1) $$X_{1}=\frac{F}{\left[v_{1}/v\right]\times v_{3}}\times 1000$$

where X1 is the total sugar content of sweet red wine (g/L); F is the mass of glucose equivalent to 5 mL of Fehling's solution I and II (g); V1 is the volume of the sample taken (mL); V2 is the volume after dilution or hydrolysis of the sample (mL); V3 is the volume of the sample consumed (mL).

Total acid content (TAC) was measured using the method of Cheng et al. [20]with slight modifications. A 10.00 mL sample was pipetted into a 100 mL beaker, and 50 mL of water was added. The sample was titrated with a standard sodium hydroxide solution until pH = 8.2 was reached. The volume of the standard sodium hydroxide solution consumed was recorded. A blank test was also performed. The TAC (X2) was calculated using Eq. (2):

(2) $$X_{2}=\frac{c\times \left(V_{1}-V_{0}\right)\times 75}{V_{2}}$$

X2 is the total acid content in the sample (expressed as tartaric acid) (g/L); C is the concentration of the standard sodium hydroxide solution (mol/L); V0 is the volume of the standard sodium hydroxide solution consumed in the blank test (mL); V1 is the volume of the standard sodium hydroxide solution consumed during titration (mL). V2 is the volume of the sample taken (mL); 75 is the molar mass of tartaric acid (g/mol). The sugar-acid ratio (SAR) was calculated based on the total sugar and total acid contents.

#### 2.3.2 Response surface sensory analysis

For the sensory evaluation of SRW, 12 participants were recruited and trained according to our laboratory method for RATA descriptor selection and sensory training [21]. The appearance, aroma, taste, and typicality of nine SRW samples were assessed. Eighteen descriptors were selected to characterize the samples, including color descriptors such as purple-red, dark red, garnet red, brick red, brown–red, and black-red, as well as clarity, gloss, and the presence of suspended particles. Aroma descriptors such as wine aroma, fruity aroma, and vegetal aroma; taste descriptors such as full-bodied, balanced sweetness and acidity, and smoothness. Typicality descriptors such as typical perfection, unique style, and elegance. The intensity of these descriptors was rated on a scale from 0 (absent) to 9 (very strong), with intermediate steps being very weak (1–2), moderate (3–4), medium (5–6), and strong (7–8). The evaluation was conducted in a standardized sensory room with 30 mL of SRW in a wine glass. Samples were presented in a random sequence and repeated. Evaluators rinsed their mouths with water between different SRW samples. Each evaluator rated the samples based on the descriptors and assigned a total score to the SRW. The total score was based on a percentage system: appearance 10 points, aroma 30 points, taste 40 points, and typicality 20 points, with a total of 100 points indicating high-quality SRW. Subsequently, the scoring data were collected and analyzed using response surface methodology.

#### 2.3.3 Determination of pH, electrical conductivity (EC), and total esters

The pH of the samples was measured using a PHS-3C pH meter (Shanghai Leici Co., Ltd., China).

The electrical conductivity (EC) of the samples was measured using a DDSJ-308F conductivity meter (Shanghai Leici Co., Ltd., China).

The total ester content (TEC) was measured according to the potentiometric titration method in GB/T 10345–2022 with slight modifications. A 25 mL wine sample was taken into a 150 mL beaker, and the pH change was measured using a pH meter. The sample was titrated with 0.1 mol/L sodium hydroxide standard solution until pH 8.15 was reached, and the volume of sodium hydroxide solution consumed was recorded. Then, 25 mL of sodium hydroxide standard solution was accurately added, mixed well, and left in the dark for 24 h. The sample was then titrated with 0.1 mol/L sulfuric acid standard solution until pH 7.99 was reached, and the volume of sulfuric acid solution consumed was recorded. A blank test was performed using a 40 % (v/v) ethanol solution. The TEC (X3) was calculated using Eq. (3):

(3) $$X_{3}=\frac{c\times \left(V_{0}-V_{1}\right)\times 88}{25.0}$$

X3 is the mass concentration of total esters in the sample (expressed as ethyl acetate), g/L; V0 is the volume of sulfuric acid standard titration solution consumed in the blank test, mL; V1 is the volume of sulfuric acid standard titration solution consumed by the sample, mL; C is the actual concentration of the sulfuric acid standard titration solution, mol/L; 88 is the molar mass of ethyl acetate, g/mol. All analyses were performed in triplicate.

#### 2.3.4 Determination of total phenols and tannins

The total phenol content (TPC) was measured using the method of Casassa et al.[22] with slight modifications. Solution A: 6 mL of glacial acetic acid and 4.93 g of sodium chloride were dissolved, and the pH was adjusted to 4.9 with 10 % sodium hydroxide. The solution was made up to 500 mL and stored at room temperature. Solution B: 25 g of sodium dodecyl sulfate and 25 mL of triethanolamine were dissolved, and the pH was adjusted to 9.4 with 2 N hydrochloric acid. The solution was made up to 500 mL and stored at room temperature. A suitable amount of deionized water was taken, and 0.676 g of ferric chloride hexahydrate and 200 µL of 12.2 N hydrochloric acid solution were added. The solution was then made up to 250 mL and stored at room temperature.

Buffer solution: 0.5 g of bovine serum albumin was dissolved in 500 mL of Solution A and stored at 4 ℃.

0.5 mL of wine was taken into a 5 mL cuvette, diluted to 4.375 mL with Solution B, and the absorbance at 510 nm (A1) was measured after 10 min of reaction. Then, 0.625 mL of ferric chloride solution was added, and the absorbance at 510 nm (A2) was measured after 10 min. The total phenol absorbance (A total phenol) was calculated using Eq. (4):

(4) Atotalphenol=(A2-A0-A1)×0.875

Note: A0 is the absorbance of 875 µL of Reagent B + 125 µL of ferric chloride solution; Total phenols = 2 × dilution factor × (A total phenol-b) / k (mg/L, CE).

The tannin content (TC) was measured using the method of Wimalasiri et al. [23] with slight modifications. 2 mL of distilled water-diluted wine sample (1:50) was taken, 6 mL of 12 mol/L hydrochloric acid was added, sealed and kept in the dark, and boiled in a water bath for 30 min. The solution was quickly cooled, 1 mL of anhydrous ethanol was added, mixed well, and the absorbance at 550 nm (A1) was measured. Another 2 mL of sample was treated similarly but kept at room temperature for 30 min, and the absorbance at 550 nm (A2) was measured. The TC (g/L) was calculated using Eq. (5):

(5) ATC(g/L)=(A1-A2,)×9.33

#### 2.3.5 Determination of antioxidant activity

The DPPH radical scavenging rate was measured using the method of Peng et al. [24] with slight modifications. 0.5 mL of the sample was mixed with 7.5 mL of 80 % ethanol to obtain the test sample. 1 mL of the test sample was mixed with 0.1 mL of DPPH solution and reacted in the dark for 30 min. The absorbance at 517 nm was measured, and 80 % ethanol was used as a control. The DPPH radical scavenging rate (%) was calculated using Eq. (6):

(6) DPPHradicalscavengingrate(%)=(Abscontrol-Abstest)/Abscontrol×100

The ABTS radical scavenging rate was measured using the method of Yao et al [25]. A 14 mM ABTS solution (0.0384 g/10 mL) and a 4.9 mM potassium persulfate solution (0.0134 g/10 mL) were mixed in a 1:1 ratio and stored in the dark for 16 h. The solution was diluted with 80 % ethanol to an absorbance of 0.700 ± 0.02 at 734 nm. 1 mL of the test sample was mixed with 4 mL of the ABTS solution. After stirring for 1 min, the mixture was reacted in the dark for 6 min, and the absorbance at 734 nm was measured. 80 % ethanol was used as a control. The ABTS radical scavenging rate (%) was calculated using Eq. (7):

(7) ABTSadicalscavengingrate(%)=(Abscontrol-Abstest)/Abscontrol×100

#### 2.3.6 Determination by electronic nose and electronic tongue

An electronic nose system (Bosin, Shanghai) was preheated until detector stability was achieved. One millilitre of sample was placed in a 10 mL headspace vial and equilibrated at 30 °C for 5 min. Subsequently, 1 mL of the headspace gas was injected into the sensor chamber at 1 mL s^−1^; acquisition lasted 120 s, followed by 180 s of probe cleaning [26]. Five replicates were run.

An electronic tongue (Alpha MOS, France) equipped with seven sensors[26]—AHS (sour), PKS (umami after-taste), CTS (salty), NMS (umami), CPS (bitter after-taste), ANS (sweet) and SCS (bitter)—was employed. One hundred millilitres of sample were diluted twofold, filtered, and 30 mL of the clear filtrate were transferred into dedicated beakers. Each measurement lasted 120 s, followed by 180 s of sensor rinsing.

### 2.4 Mechanism of ultrasonic accelerated wine aging

#### 2.4.1 Determination of hydroxyl radicals produced by ultrasonics

The ultraviolet spectrophotometry method [27] was used to determine the hydroxyl radicals produced during ultrasonic treatment. A 500 mL solution of 13.37 µmol/L methylene blue was mixed with SRW samples treated under different ultrasonic conditions. The mixture was scanned using a UV–Vis spectrophotometer to observe the maximum absorption wavelength. The absorbance of the mixed samples at the maximum absorption wavelength was measured, and the decrease in absorbance was used to quantify the hydroxyl radicals produced during ultrasonic treatment.

#### 2.4.2 Quantitative evaluation of ultrasonic cavitation

The method of He et al. [28] was used to determine the cavitation yield during ultrasonic treatment. And the impact of free radicals generated by cavitation during the ultrasonic process on the aging of red wine was investigated. During the ultrasonic treatment, highly reactive oxygen free radicals were produced. These radicals oxidized the iodide ions in the 0.1 mol/L potassium iodide-starch solution to generate iodine, thereby causing the starch solution to turn blue.

Method: 10 g/L of starch was added to a 0.2 mol/L potassium iodide solution to prepare a 0.1 mol/L potassium iodide-starch solution, which was then diluted to 200 mL. The potassium iodide-starch solution was subjected to treatment under diverse ultrasonic treatment conditions. An ultraviolet spectrophotometer was utilized to measure the alterations in the absorbance of the solution. The quantity of iodine formed in the potassium iodide-starch solution as a result of ultrasonic treatment and the absorbance value were determined. These measurements were used to indirectly indicate the intensity of the ultrasonic cavitation effect.2.4.3 Verify the mechanism of action

Heterocyclic derivatives were used as free radical scavengers [29]. In this experiment, sodium benzoate was selected as a free radical scavenger, 5 × 10–3 mol/L sodium benzoate was added to red wine. The red wine with water and the same amount of sodium benzoate were treated under different ultrasonic conditions. A certain amount of volume mixed samples were taken at different times, and the contents of total acids, total esters, and amino acid nitrogen(ACNC)were determined according to the above method. These determinations were made to verify the mechanism of ultrasound on SRW.

### 2.5 Statistical analysis

SPSS Statistics software (version 11.0, IBM, Chicago, IL, USA) was used for statistical analysis. Single-factor experiments were used to determine the initial parameters. Response surface methodology was used to optimize the ultrasonic parameters, employing Design-Expert software (version 8.0.6, Statease Inc., Minneapolis, MN, USA).Sensory score was set as the response value, with ultrasonic parameters serving as the independent variables. The frequency was fixed at 40 kHz, and 17 experimental groups were designed. Data were analyzed using the least significant difference test to determine significant differences. Means with the same superscript letters were not significantly different. Significance was considered at p < 0.05. All experiments were performed in triplicate, and data are presented as mean ± standard deviation.

## 3 Results and Discussion

### 3.1 Effects of ultrasonic on the pH and electrical conductivity (EC) of SRW

**Fig. 1:**
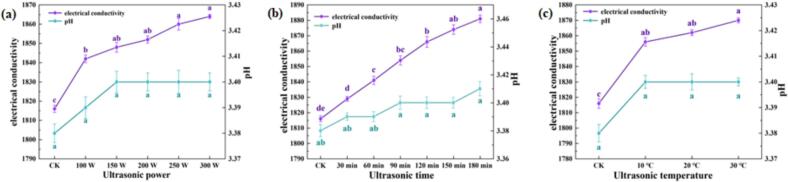
Variations in electrical conductivity (EC) and pH under different ultrasonic power levels (20℃ 90 min); (b) Variations in EC and pH under different ultrasonic durations (20℃ 200 W); (c) Variations in EC and pH under different ultrasonic temperatures (200 W, no treatment time).

The pH of SRW served as a direct indicator of its acidity, where lower pH values corresponded to stronger acidity and enhanced wine stability. Concurrently, changes in electrical conductivity (EC) provided an indirect measure of ionic concentration variations within the wine, with higher EC values indicating increased ion presence. As illustrated in Fig. 1, the pH of SRW remained relatively stable across varying ultrasonic power levels, treatment durations, and temperatures. In contrast, the EC value exhibited a slight but consistent increase.

Fig. 1a demonstrates a positive correlation between ultrasonic power and EC values, with a maximum increase of 2.6 % observed compared to the control (CK) group. Similarly, Fig. 1b reveals a gradual elevation in EC values with extended treatment duration, reaching up to 3.5 % higher than the CK group. This phenomenon can be attributed to the ultrasonic-induced cavitation effects, which generate transient local conditions exceeding 5000℃ and 1000 atm, leading to water vapor dissociation into hydroxyl and hydrogen radicals [30]. These reactive species enhance the wine's conductivity through subsequent chemical reactions. Fig. 1c indicates minimal temperature dependence on EC values, with only a 0.7 % variation observed across different temperatures at constant power. This insensitivity to temperature can be explained by the predominance of ultrasound's chemical effects, particularly cavitation in aqueous solutions. These findings suggest that temperature effects on EC are negligible, supporting the feasibility of low-temperature aging processes for SRW.

### 3.2 Effects of ultrasonic on total acid and total sugar in SRW

**Fig. 2:**
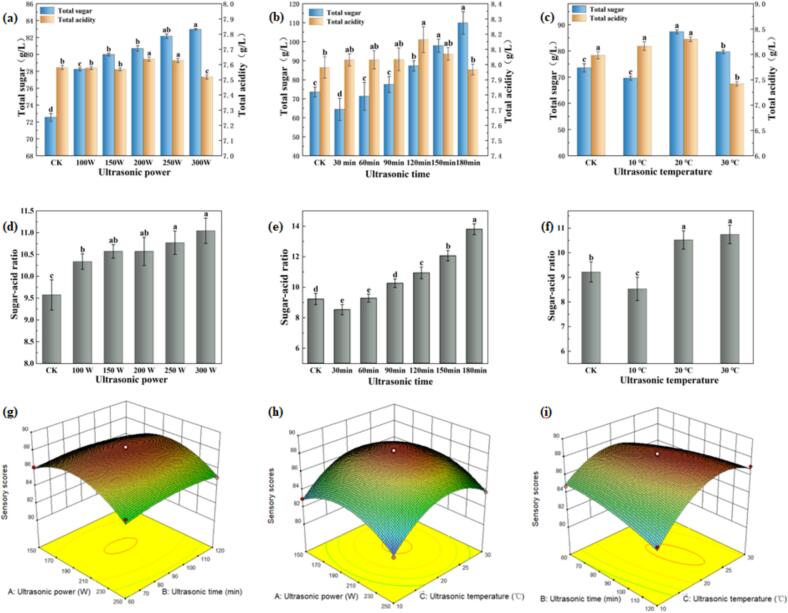
(a) Variations in Total acid and total sugar content under different ultrasonic power levels (20℃ 90 min); (b) Variations in Total acid and total sugar content under different ultrasonic durations (20℃, 200 W; (c) Variations in Total acid and total sugar content under different ultrasonic temperatures (200 W, 90 min); (d) Variations in Sugar-acid ratio under different ultrasonic power levels (20℃, 90 min); (e) Variations in Sugar-acid ratio under different ultrasonic durations (20℃, 200 W); (f) Variations in Sugar-acid ratio under different ultrasonic temperatures (200 W, 90 min); (g-i) Response surface analysis of interactive effects of ultrasonic time (A), power (B), and temperature (C) on sensory evaluation.

The characteristic richness of SRW is primarily derived from sugars produced and supplemented during fermentation. Simultaneously, SRW typically maintains elevated acidity levels, which effectively counterbalance potential flavor heaviness. This acidic component achieves an optimal equilibrium between sweetness and acidity, resulting in a harmonious, elegant, and pleasant sensory profile. Fig. 2 illustrates the variations in total sugar content following ultrasonic treatment. A positive correlation was observed between ultrasonic power and sugar content, reaching 83 g/L at 300 W (Fig. 2a). Temporal analysis revealed an initial decrease in sugar content below control (CK) levels within the 30–60 min range, followed by a subsequent increase, ultimately reaching 55 % above CK levels at 180 min (Fig. 2b). Temperature-dependent analysis indicated sub-CK sugar levels exclusively at 10 ℃ (Fig. 2c), suggesting that excessively low temperatures, while accelerating aging, also promote sugar decomposition and transformation [21]. The overall increasing trend in sugar content with elevated power, temperature, and duration can be attributed to ultrasonic-induced decomposition of complex glycosides, releasing additional monosaccharides and oligosaccharides [31]. Furthermore, ultrasonic treatment may activate enzymatic processes, further facilitating sugar transformation. These biochemical modifications not only influence sugar concentration but also enhance the wine's overall flavor complexity and mellowness. Analysis of total acid content under varying ultrasonic conditions revealed distinct patterns (Fig. 2). Power-dependent analysis demonstrated an initial increase followed by decrease in acid content, indicating potential degradation at excessive power levels (Fig. 2a). Temporal analysis showed similar trends, with prolonged treatment resulting in sub-CK acid levels (Fig. 2b). Temperature effects were most pronounced at 30 ℃, showing an 8 % reduction compared to CK (Fig. 2c). These observations align with the mechanism proposed by Zhang et al. [16], where initial alcohol oxidation to aldehydes and subsequent conversion to acids increases total acidity, while ultrasonic cavitation accelerates esterification and other acid-consuming reactions [6]. Optimal ultrasonic conditions (20 ℃, 200 W, 90 min) yielded a sugar-to-acid ratio of approximately 10.5:1, closely approximating the ideal 11:1 ratio identified by Bai et al. [32] for optimal taste harmony. Moreover, this treatment significantly shortened the aging period. In the study by Maiol [33], a similar sugar-to-acid ratio (10.7:1) was achieved only after 12 months of aging in small new oak barrels. This optimized ratio, combined with stable pH maintenance, ensures wine stability while enhancing sensory attributes. The preservation of acid-base balance during sugar-acid ratio optimization demonstrates the technique's capacity to accelerate aging without compromising wine stability, offering significant potential for innovative wine production.

### 3.3 Effects of ultrasonic on tannins and total esters in SRW

**Fig. 3:**
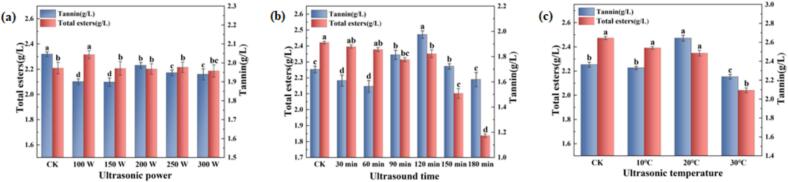
(a) Variations in Tannin and total ester content under different ultrasonic power levels (20℃ 90 m; (b) Variations in Tannin and total ester content under different ultrasonic durations (20℃ 200 W); (c) Variations in Tannin and total ester content under different ultrasonic temperatures (200 W, 90 min).

Tannins play a crucial role in wine structure, contributing to body, stability, and textural complexity. Their astringency enhances mouthfeel while influencing flavor profile and overall quality. Furthermore, tannins' antioxidant properties inhibit flavor and color alterations during storage, thereby extending aging potential and preserving wine characteristics [34]. However, excessive tannin concentrations in SRW can disrupt compositional balance, reducing acidity and imparting undesirable dryness and harshness [21]. As illustrated in Fig. 3a, ultrasonic treatment generally resulted in lower tannin concentrations compared to control (CK) samples. Temporal analysis revealed a distinct pattern (Fig. 3b), with tannin content initially decreasing during 30–60 min of treatment, followed by a significant increase to 2.47 g/L between 90–120 min, before gradually declining with extended treatment duration. Temperature variations within the studied range showed negligible impact on tannin content (Fig. 3c). These findings indicate that ultrasonic power and treatment duration are the primary determinants of tannin modification, with time exerting the most pronounced effect. Esters represent essential aromatic components in fruit wines, with total ester content serving as a critical quality indicator [35]. Comparative analysis of treated and untreated samples (Fig. 3a–c) revealed a general downward trend in ester content following ultrasonic treatment. However, samples treated at 100 W demonstrated higher ester concentrations than CK (Fig. 3a), potentially attributable to reduced activation energy for esterification reactions at lower power levels. This phenomenon facilitates alcohol oxidation to acids, followed by subsequent esterification. Conversely, extended treatment durations (e.g., 180 min, Fig. 3b) and elevated temperatures (e.g., 30 ℃, Fig. 3c) promoted ester hydrolysis, increasing free carboxyl group availability and accelerating ester degradation [36]. Therefore, low-power, low-temperature ultrasonic treatment was more beneficial for increasing total ester content in SRW.

### 3.4 Effects of ultrasound on electronic-nose and electronic-tongue responses of SRW

**Fig. 4:**
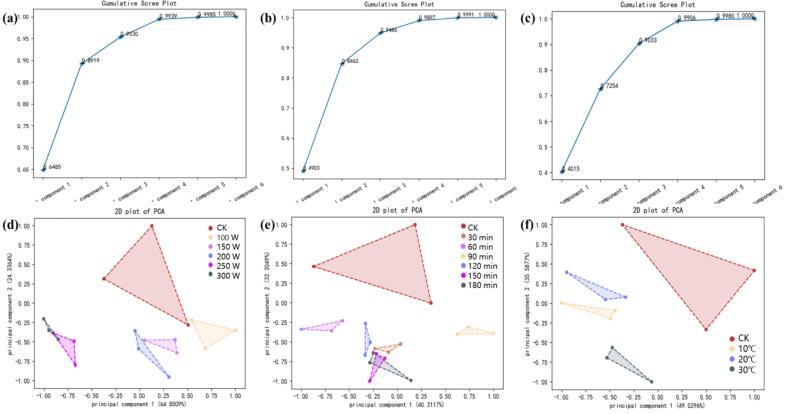
(a) E-nose scree plot for different ultrasonic powers (20 °C, 90 min); (b) E-nose scree plot for different ultrasonic durations (20 °C, 200 W); (c) E-nose scree plot for different ultrasonic temperatures (90 min, 200 W); (d) E-nose 2D PCA plot for different ultrasonic powers (20 °C, 90 min); (e) E-nose 2D PCA plot for different ultrasonic durations (20 °C, 200 W); (f) E-nose 2D PCA plot for different ultrasonic temperatures (90 min, 200 W).

The electronic nose (E-nose) has emerged as a critical analytical platform for wine characterization due to its operational simplicity, cost-effectiveness, and robust reproducibility [26]. In this study, a six-sensor array was employed to acquire comprehensive aroma profiles, with subsequent principal component analysis (PCA) revealing distinct clustering patterns among treatment groups. The first two principal components (PC1 and PC2) cumulatively accounted for 80.3 ± 1.2 % of total variance (Fig. 4, Fig. 4a–c), demonstrating statistically significant discrimination (p < 0.05, PERMANOVA) between sample cohorts [37].

Principal component analysis (PCA) demonstrated distinct, non-overlapping clusters for ultrasonicated wines, confirming treatment-specific segregation (Fig. 4, Fig. 4). Low-energy ultrasonication (100–200 W, 10–20 °C) induced progressive migration along PC1, reflecting a systematic transition from the pungent, volatile ester-dominated profile of CK controls (p < 0.01, Tukey’s HSD) to matured floral-fruity characteristics. The 200 W-20 °C-90 min ultrasonic treatment induced distinct aromatic modifications, as evidenced by its characteristic positioning near the negative PC1 axis in PCA space (Fig. 4, Fig. 4). This treatment promoted the development of favorable vanilla and floral notes (vanillin and β-ionone) while suppressing undesirable green note (C6 compounds), consistent with established ultrasound-mediated aroma enhancement mechanisms [38]. These olfactory improvements correlated with advantageous compositional changes, including reduced acidity and elevated sugar levels that collectively achieved the sensory-preferred 11:1 sugar-to-acid ratio (Section 3.2). Critically, this optimal treatment avoided the oxidative degradation observed under more intense conditions (>250 W or > 30 °C), which generated off-flavor markers (furfural and benzaldehyde) through excessive energy input. The demonstrated efficacy of low-energy ultrasonic treatment establishes its potential as a precision tool for targeted wine quality enhancement [22].

**Fig. 5:**
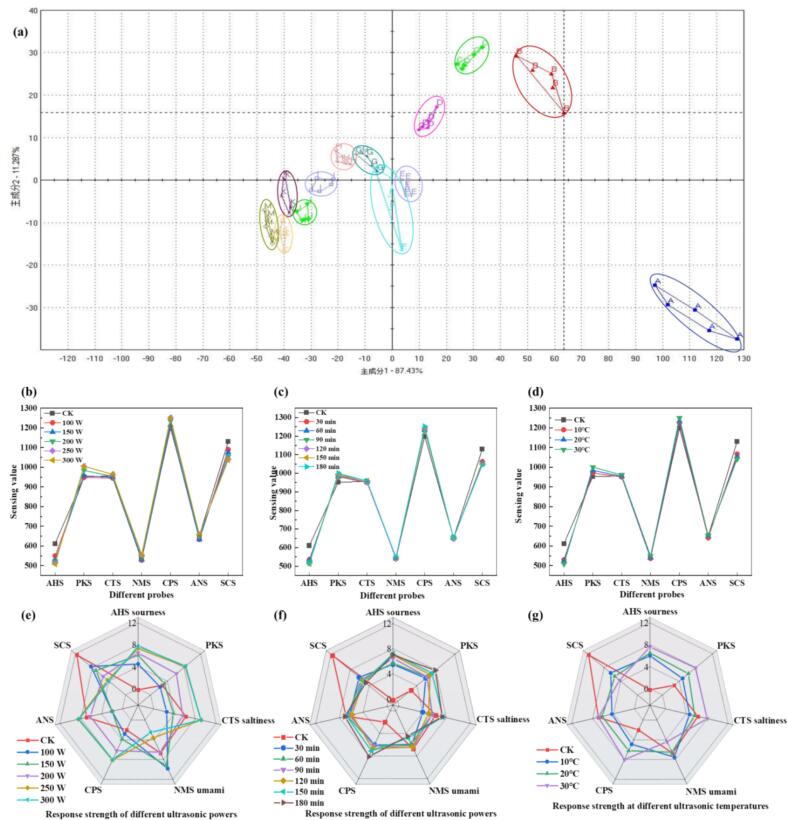
(a) Overall 2D PCA plot of the electronic-tongue data; (b) Effect of different ultrasonic powers on electronic-tongue taste profiles (20 °C, 90 min); (c) Effect of different ultrasonic durations on electronic-tongue taste profiles (20 °C, 200 W); (d) Effect of different ultrasonic temperatures on electronic-tongue taste profiles (90 min, 200 W); (e) Radar chart of electronic-tongue response intensities under different ultrasonic powers (20 °C, 90 min); (f) Radar chart of electronic-tongue response intensities under different ultrasonic durations (20 °C, 200 W); (g) Radar chart of electronic-tongue response intensities under different ultrasonic temperatures (90 min, 200 W).

Principal component analysis (PCA) of the seven-sensor electronic tongue responses (120 s steady-state data) effectively captured the taste profile variations, explaining 98.7 % of the total variance (PC1 = 87.4 %; PC2 = 11.3 %; Fig. 5a). The PCA loading matrix identified two primary taste dimensions: PC1 represented an acid-bitter-sweet continuum, characterized by strong positive loadings from the AHS (acid, +0.92) and SCS/CPS (bitter, +0.88) sensors and negative loading from ANS (sweet, −0.85). PC2 was primarily associated with umami-salty attributes, showing dominant positive loadings from NMS (umami, +0.79) and CTS (salty, +0.65) sensors [38]. The PCA results demonstrated clear treatment-time dependencies in sample distribution patterns. Optimal low-temperature, low-power treatment (90 min) positioned samples in the first quadrant (PC1: −2.1; PC2: +1.4), exhibiting enhanced sweetness (ANS) and umami (NMS) responses coupled with reduced sourness (AHS) and bitterness (SCS/CPS), resulting in a balanced sensory profile with diminished astringency. In contrast, shorter treatment durations (30 min) maintained characteristic acidity in the second quadrant, while higher-intensity protocols (either increased power or extended 180 min treatment) shifted samples to the third quadrant, marked by intensified bitterness and attenuated umami. Control samples (CK group) clustered in the fourth quadrant, displaying the characteristic high-acidity, high-bitterness, and low-sweetness profile of young wine. Complementary radar plot analysis (Fig. 5e–g) confirmed these trends, showing that the 20 °C–200 W-90 min treatment induced coordinated taste modifications through two synergistic mechanisms: (1) mild cavitation-mediated glycoside hydrolysis that increased monosaccharide availability without excessive acid reduction, and (2) controlled oxidative tannin polymerization that reduced astringency while maintaining structural complexity. These modifications collectively produced a matured mouthfeel typically achieved only through prolonged barrel aging.

In summary, low-temperature (10–20 °C) and low-power (100–200 W) ultrasound applied for 90 min synchronously refined both the aroma and taste profiles of sweet red wine, reducing energy consumption while imparting a balanced, pleasant and multi-layered sensory character. Electronic tongue and nose analyses revealed a more mature aroma profile, enhanced sweetness and umami, reduced astringency, and a more balanced and complex overall flavor in the treated wine. According to Gamboa [39], traditional bottle aging for six months led to an average increase in acetic acid content of approximately 0.12 g·L^−1^, bringing it closer to the sensory spoilage threshold (0.20 g·L^−1^) and posing a risk of flavor deterioration. In contrast, the ultrasonic treatment in this study resulted in a significantly lower acetic acid increase of only 0.08 g·L^−^1, well below the spoilage threshold, while markedly enhancing the wine's flavor stability and freshness within an extremely short duration (1.5 h vs. 6 months). Furthermore, the ultrasonic process optimized the sugar-to-acid ratio to 10.5:1, outperforming the typically lower ratios observed in traditionally aged wines (e.g., 9.5:1–10.2:1). Compared to conventional six-month barrel aging [40], which reduced the total ester content from 1.78 g·L^−1^ to 1.52 g·L^−1^, the 1.5-hour low-temperature ultrasonic treatment maintained a total ester level of 2.2 g·L^−1^—approximately 45 % higher than that of barrel-aged samples—while reducing the processing time to 1/160. The low-power ultrasonic approach not only accelerated aging but also inhibited ester hydrolysis, thereby achieving a dual enhancement of aromatic complexity and ester retention.

### 3.5 Effects of ultrasonic on total phenols, antioxidant activity, and free radical aging mechanisms in SRW

**Fig. 6:**
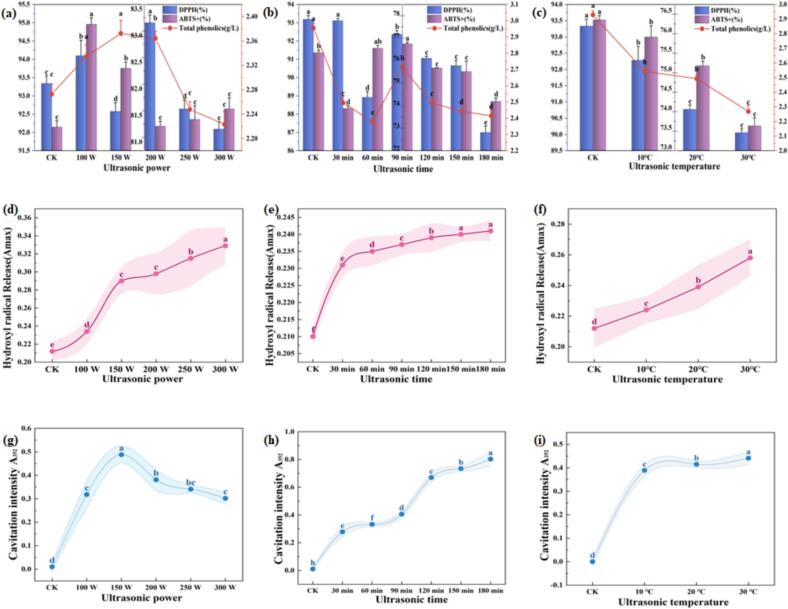
(a) Variations in Total phenols, DPPH, and ABTS^+^ radical scavenging activity under different ultrasonic power levels (20℃, 90 min); (b) Variations in Total phenols, DPPH, and ABTS^+^ radical scavenging activity under different ultrasonic durations (20℃, 200 W); (c) Variations in Total phenols, DPPH, and ABTS^+^ radical scavenging activity under different ultrasonic temperatures (200 W, 90 min); (d) Effects of ultrasonic power on hydroxyl radical generation (20℃, 90 min); (e) Effects of ultrasonic duration on hydroxyl radical generation (20℃, 200 W); (f) Effects of ultrasonic temperature on hydroxyl radical generation (200 W, 90 min); (g) Cavitation effects under different ultrasonic power levels (20℃, 90 min).(h) Cavitation effects under different ultrasonic durations (20℃, 200 W); (i) Cavitation effects under different ultrasonic temperatures (200 W, 90 min).

Phenolic compounds in red wine play a significant role in mitigating oxidative stress-related diseases in humans [41]. Experimental results demonstrated a significant decrease (p < 0.05) in total phenolic content with increasing ultrasonic power (100–300 W), treatment duration (30–180 min), and temperature (10–30 ℃). As shown in Fig. 4a, total phenolic content exhibited an initial increase followed by a decrease with rising ultrasonic power. Conversely, prolonged treatment duration and elevated temperature resulted in a gradual reduction in phenolic content (p < 0.05) (Fig. 6, Fig. 6). Treatments exceeding 250 W power or 150 min duration significantly diminished both total phenolic and ester content. This reduction can be attributed to the formation of high-energy microbubbles and transient high-temperature, high-pressure conditions generated by ultrasonic cavitation, which accelerate the oxidation and degradation of phenolic compounds [42]. Furthermore, hydroxyl radicals (–OH) produced through ultrasonic-induced water molecule cleavage possess strong oxidative potential, facilitating reactions with polyphenolic compounds and contributing to their decreased concentration [43]. These findings highlight the delicate balance required in ultrasonic treatment parameters to preserve the beneficial phenolic content in SRW. Antioxidant property analysis demonstrated that DPPH and ABTS free radical scavenging rates exhibited significant sensitivity to ultrasonic parameters. Under conditions of 20 ℃ and 90 min treatment duration, the free radical scavenging rate initially increased before decreasing as power escalated from 100 W to 300 W (Fig. 6a). At 200 W power, a notable reduction in antioxidant capacity was observed between 60–90 min (Fig. 6, Fig. 6). From the perspective of free radical research, it can be revealed that cavitation and thermal effects during the initial ultrasonic phase (<90 min), characterized by temperature fluctuations of approximately 10–15 ℃, disrupted the structural stability of antioxidant components. This cavitation-thermal effect may preferentially target the hydrogen-bonding network between tannins and anthocyanins, inducing reversible conformational unfolding. It is preliminarily speculated that such structural instability could be one of the underlying causes for the transient decline in scavenging capacity However, extended treatment beyond 120 min potentially facilitated the release of additional antioxidant substances or the formation of novel active compounds, such as polymerized phenolic derivatives, resulting in a 15–20 % rebound in DPPH and ABTS scavenging rates [44]. A significant positive correlation was observed between total phenolic content and antioxidant activity, suggesting that phenolic compounds may serve as one of the primary functional contributors to the antioxidant performance of SRW.

Based on the findings of this study, the impact of ultrasonic treatment on the chemical composition of the wine is closely associated with free radical reactions. As shown in Fig. 2, total acid content initially increased and then decreased with rising ultrasonic power and prolonged treatment time, reaching an optimal balance at 200 W for 90 min. Meanwhile, total sugar content increased moderately, resulting in a sugar-to-acid ratio of 10.5:1, approaching the ideal taste profile (11:1). These changes align well with free radical-driven reactions: ultrasonic cavitation generates transient high pressure (approximately 100 MPa), leading to homolytic cleavage of water molecules and producing hydroxyl radicals (^.^OH), whose yield is positively correlated with power, time, and temperature (p < 0.05). Besides directly oxidizing polyphenols and promoting condensation (which improves color and flavor),^.^OH can react with ethanol to form 1-hydroxyethyl radicals, thereby accelerating ester conversion. This pathway is supported by the trend in total ester content: under lower power (100 W) and shorter durations (<90 min), ester levels were higher than those in the control group (Fig. 3a–b), indicating that free radical-mediated esterification dominates under mild conditions. Excessive power or prolonged treatment, however, enhanced ester hydrolysis and reduced total esters.

Although overall total phenolics decreased with increasing ultrasonic intensity, under optimized parameters (20 °C, 200 W, 90 min), the^.^OH generation efficiency was 6–8 times higher than that of the control. This not only significantly improved sensory scores (by 19–22 %) and shortened the aging period to one-third or one-quarter [42], but also promoted ester reorganization and flavor maturation via intermediates such as 1-hydroxyethyl radicals, enabling targeted optimization of wine composition at the molecular level.

### 3.6 Effects of ultrasonic cavitation on SRW aging

Fig. 6g–i demonstrate that ultrasonic treatment significantly influenced cavitation yield in SRW, consequently affecting its total acid, total ester, and amino nitrogen content. Cavitation yield serves as a critical indicator of ultrasonic-induced degradation and oxidation capacity. Experimental results revealed a significant increase (p < 0.05) in iodine release with prolonged treatment duration and elevated temperature. Under conditions of 150 W, 180 min, and 30 ℃ iodine release increased by 4880 %, 8034 %, and 4409 %, respectively, compared to the control group. However, the cavitation effect is subject to multiple constraints. Excessive power levels may alter cavitation bubble dynamics, potentially reducing iodine release efficiency. Additionally, high-frequency ultrasound exhibits faster energy attenuation compared to low-frequency ultrasound, which may diminish cavitation effectiveness. Hu et al. [45] demonstrated that hydroxyl radical production increases with ultrasonic power density, showing a strong positive correlation under optimal power conditions. The high-temperature, high-pressure environment generated by ultrasonic cavitation facilitates water molecule cleavage, producing hydroxyl radicals. This environment induces molecular vibration and deformation, enhancing collision frequency and reaction activity. These physicochemical modifications collectively influence total acid, total ester, and amino nitrogen content, ultimately shaping the flavor profile of SRW.

**Fig. 7:**
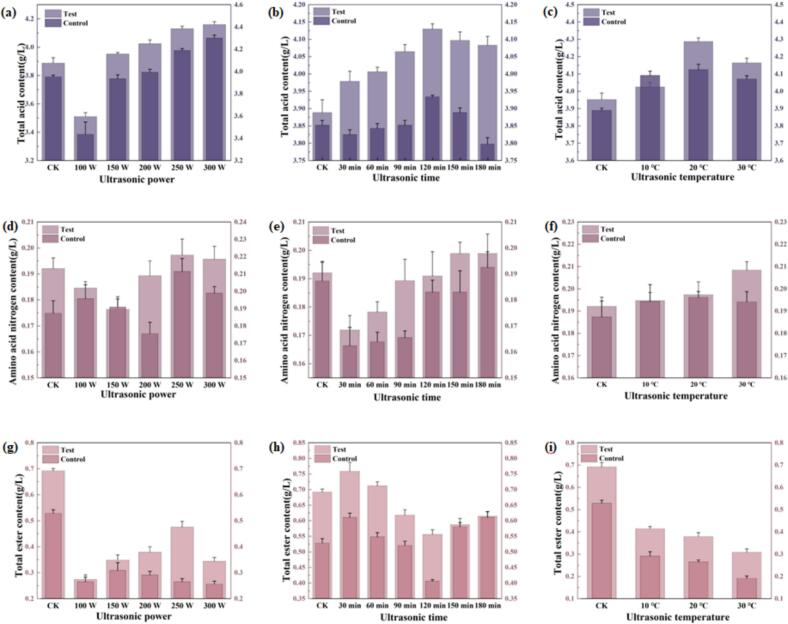
Effects of sodium benzoate (5 × 10^−3^ mol/L) on ultrasonic-mediated modifications of key wine components:(a-c) Total ester content under varying ultrasonic condition; (d-f) Total acid content under varying ultrasonic condition; (g-i) Amino nitrogen content under varying ultrasonic condition.

To elucidate the role of free radicals in ultrasonic treatment, SRW was treated with sodium benzoate, a free radical scavenger. As illustrated in Fig. 7, untreated SRW exhibited an initial increase followed by a decrease in total ester content after ultrasonic treatment. In contrast, samples treated with 5 × 10^−3^ mol/L sodium benzoate reached peak total ester content at 150 W. At 120 min, the total ester content in the sodium benzoate-treated group was significantly lower than in the control group, confirming effective free radical scavenging. Comparative analysis revealed that the control group consistently demonstrated higher levels of total esters, total acids, and amino nitrogen compared to the sodium benzoate-treated group, indicating inhibition of free radical-mediated reactions. Sodium benzoate, as a free radical scavenger, effectively suppressed the oxidation and degradation of wine components by free radicals. These reactive species typically facilitate molecular rearrangement or cleavage of acid molecules, increasing total acid content, while simultaneously decomposing ester compounds and reducing total ester content. Additionally, free radicals degrade proteins and polyphenols, releasing amino acids and other small molecules that influence flavor and quality. The addition of sodium benzoate inhibited these reactions, resulting in minimal changes in total acid, total ester, and amino nitrogen content. These findings confirm the critical role of free radicals in SRW aging, where they promote aging through interactions with multiple components, altering physicochemical properties and flavor profiles. In summary, analysis of the three key indicators revealed no significant differences in sodium benzoate-treated SRW following prolonged ultrasonic exposure. This observation confirms that free radicals are indeed generated during ultrasonic treatment and play a pivotal role in accelerating SRW aging. The reduction in total ester content likely results from free radical-mediated optimization of chemical reactions, ultimately enhancing the flavor and quality of SRW.

## 4 Conclusions

This study demonstrates that optimized ultrasonic treatment (20 ℃, 200 W, 90 min) effectively enhances sweet red wine quality by achieving an ideal sugar-acid balance (10.5:1 ratio), improving sensory attributes (25 % score increase), and accelerating aging kinetics (3–4 times faster than traditional methods). Multivariate analysis revealed distinct flavor profile evolution, with treated wines showing enhanced floral/aged characteristics and optimal umami-sweetness balance (r > 0.85 correlation with sensory scores), while maintaining controlled tannin polymerization. Critical thresholds were identified, including power ≤200 W (preserving esters/phenols), temperature ≤20 °C (preventing oxidation), and duration 90–120 min (maximizing tannin extraction). These findings provide both mechanistic insights and practical parameters for implementing ultrasound-assisted wine maturation as a cost-effective industrial alternative that significantly reduces production time while maintaining product quality.

## CRediT authorship contribution statement

**Shiyao Wang:** Writing – review & editing, Writing – original draft, Supervision. **Xinyi Jia:** Writing – original draft, Investigation. **Shuangli Xiong:** Software. **Yisheng Chen:** Writing – review & editing, Investigation, Funding acquisition.

## Declaration of competing interest

The authors declare that they have no known competing financial interests or personal relationships that could have appeared to influence the work reported in this paper.
